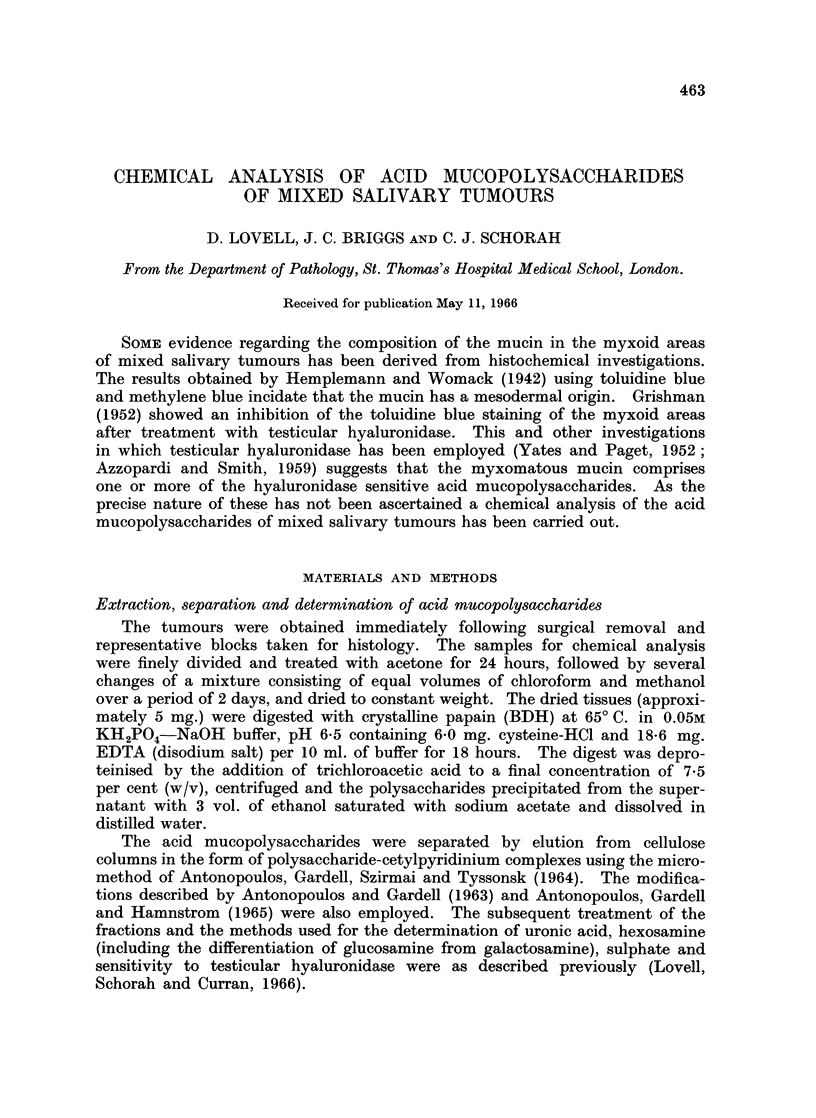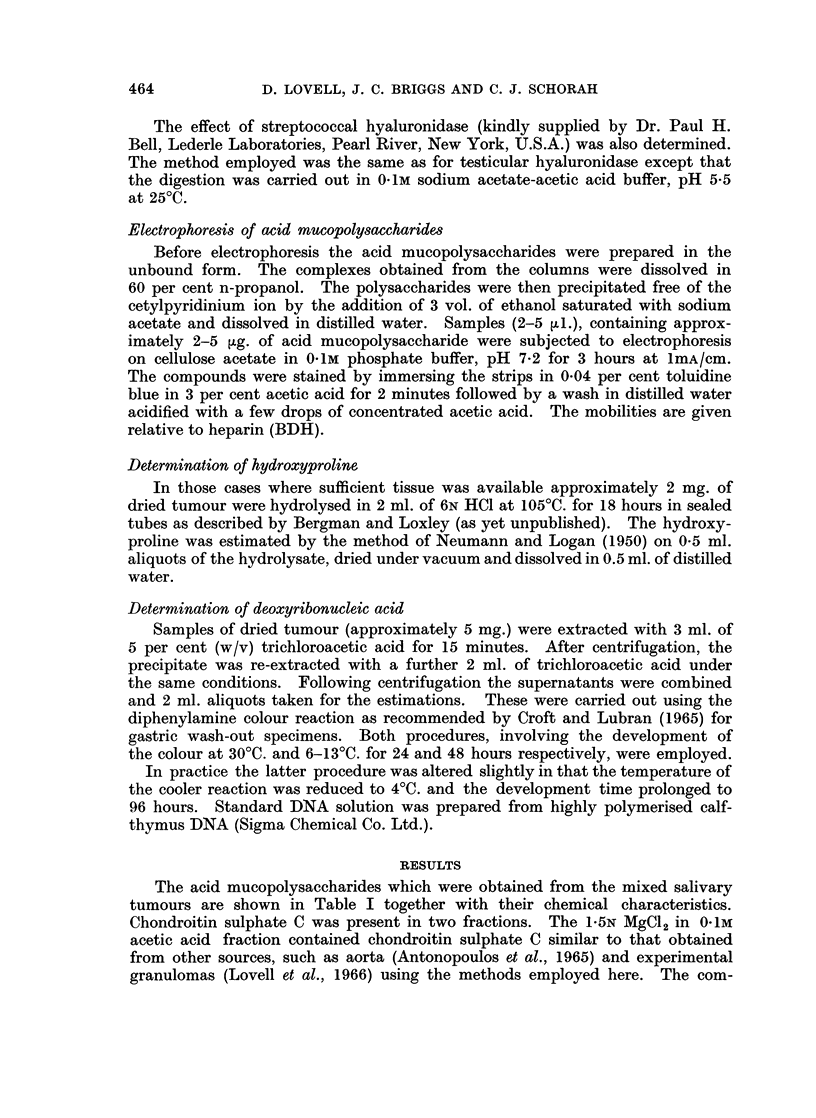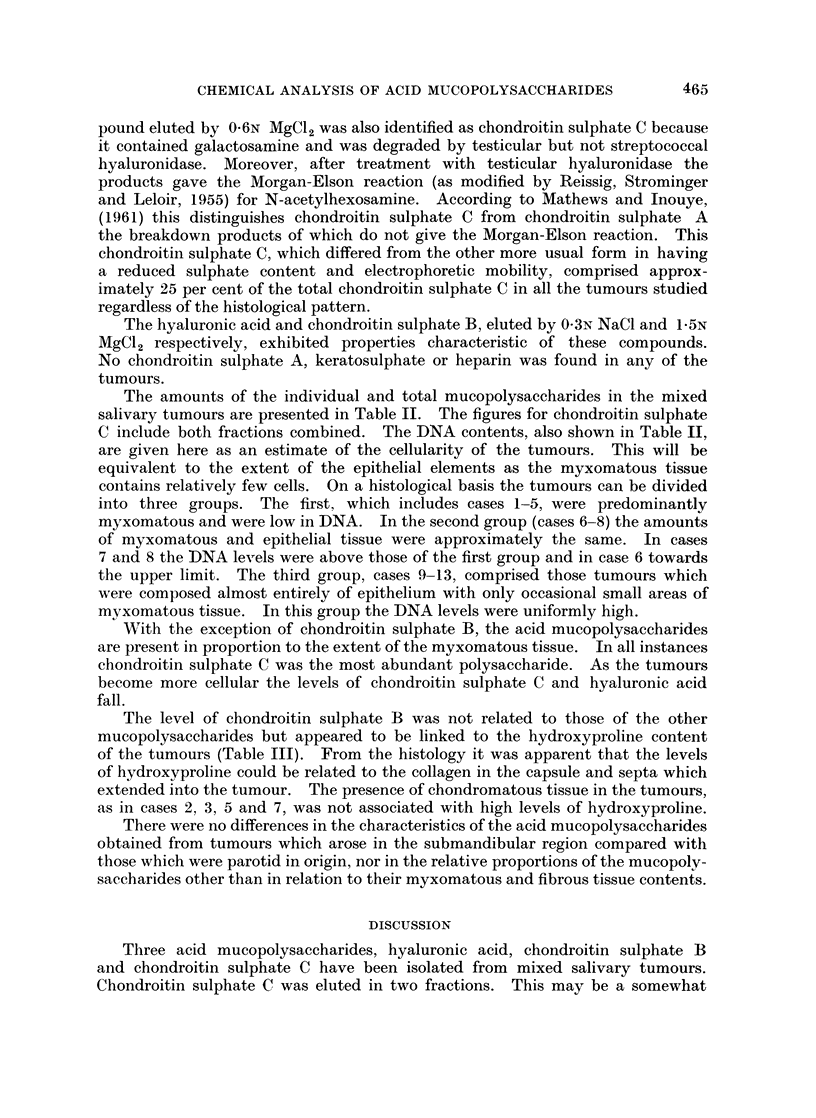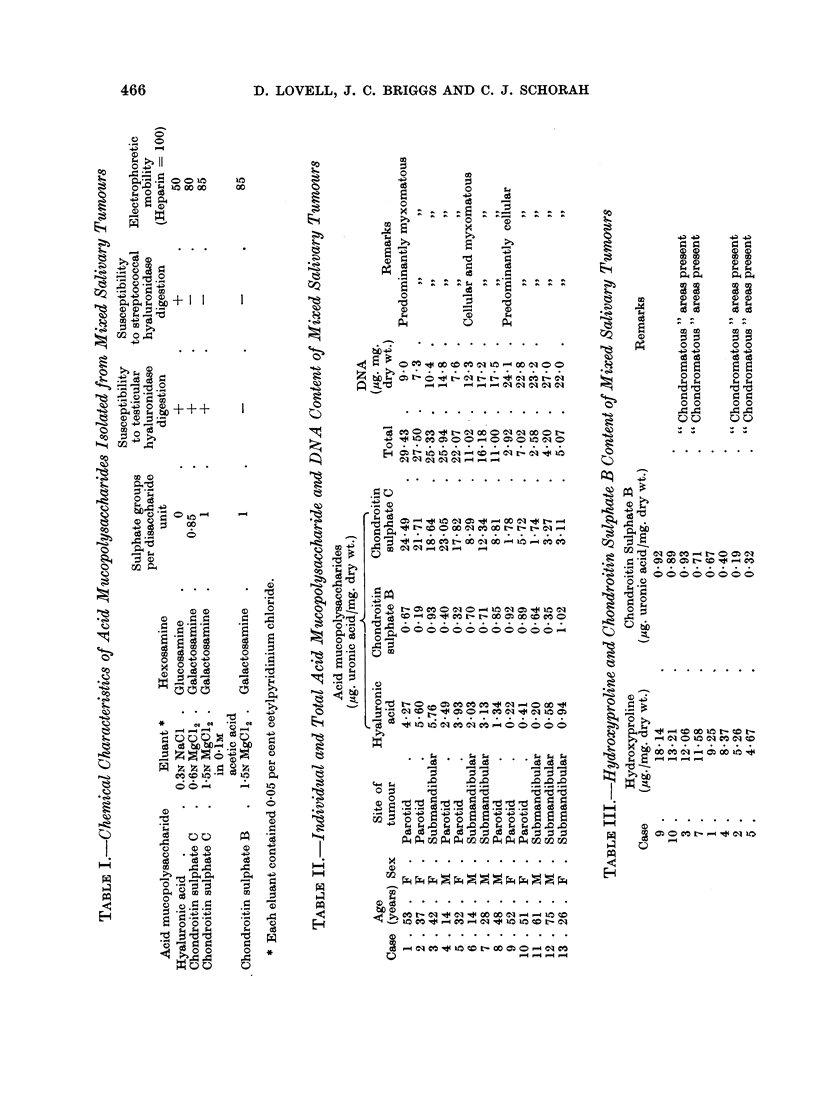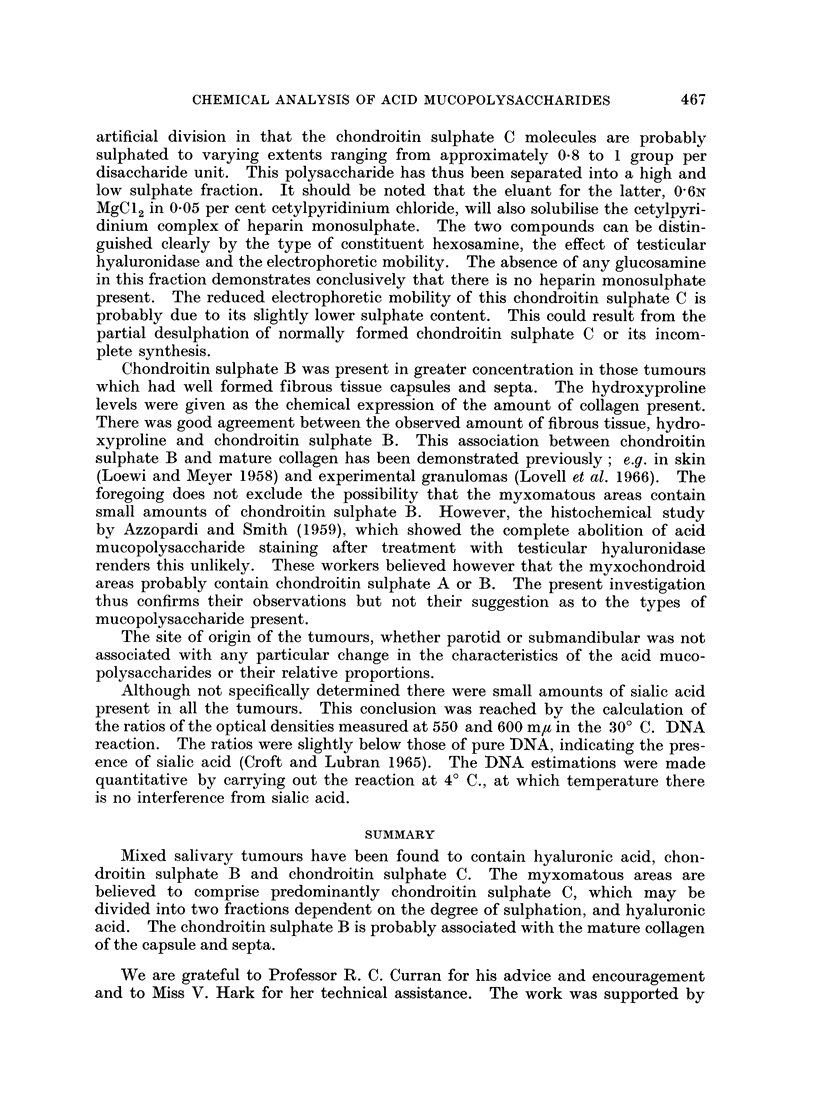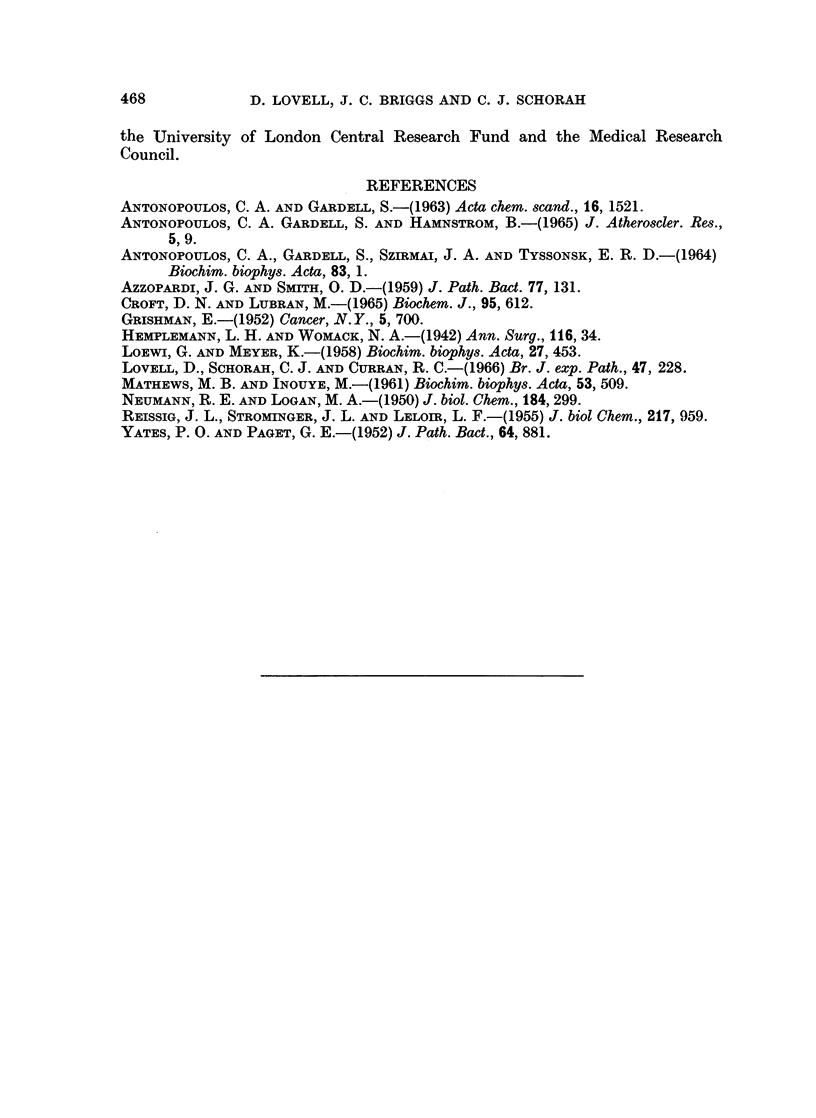# Chemical analysis of acid mucopolysaccharides of mixed salivary tumours.

**DOI:** 10.1038/bjc.1966.56

**Published:** 1966-09

**Authors:** D. Lovell, J. C. Briggs, C. J. Schorah


### CHEMICAL ANALYSIS OF ACID MUCOPOLYSACCHARIDES OF MIXED SALIVARY TUMOURS

D. LOVELL, J. C. BRIGGS AND C. J. SCHORAH

*From the Department of Pathology, St. Thomas's Hospital Medical School, London.*

Received for publication May 11, 1966

SOME evidence regarding the composition of the mucin in the myxoid areas of mixed salivary tumours has been derived from histochemical investigations. The results obtained by Hemplemann and Womack (1942) using toluidine blue and methylene blue incidate that the mucin has a mesodermal origin. Grishman (1952) showed an inhibition of the toluidine blue staining of the myxoid areas after treatment with testicular hyaluronidase. This and other investigations in which testicular hyaluronidase has been employed (Yates and Paget, 1952; Azzopardi and Smith, 1959) suggests that the myxomatous mucin comprises one or more of the hyaluronidase sensitive acid mucopolysaccharides. As the precise nature of these has not been ascertained a chemical analysis of the acid mucopolysaccharides of mixed salivary tumours has been carried out.

### MATERIALS AND METHODS

### *Extraction, separation and determination of acid mucopolysaccharides*

The tumours were obtained immediately following surgical removal and representative blocks taken for histology. The samples for chemical analysis were finely divided and treated with acetone for 24 hours, followed by several changes of a mixture consisting of equal volumes of chloroform and methanol over a period of 2 days, and dried to constant weight. The dried tissues (approximately 5 mg.) were digested with crystalline papain (BDH) at 65° C. in 0.05M $KH_2PO_4$—NaOH buffer, pH 6·5 containing 6·0 mg. cysteine-HCl and 18·6 mg. EDTA (disodium salt) per 10 ml. of buffer for 18 hours. The digest was deproteinised by the addition of trichloroacetic acid to a final concentration of 7·5 per cent (w/v), centrifuged and the polysaccharides precipitated from the supernatant with 3 vol. of ethanol saturated with sodium acetate and dissolved in distilled water.

The acid mucopolysaccharides were separated by elution from cellulose columns in the form of polysaccharide-cetylpyridinium complexes using the micromethod of Antonopoulos, Gardell, Szirmai and Tyssonsk (1964). The modifications described by Antonopoulos and Gardell (1963) and Antonopoulos, Gardell and Hamnstrom (1965) were also employed. The subsequent treatment of the fractions and the methods used for the determination of uronic acid, hexosamine (including the differentiation of glucosamine from galactosamine), sulphate and sensitivity to testicular hyaluronidase were as described previously (Lovell, Schorah and Curran, 1966).

The effect of streptococcal hyaluronidase (kindly supplied by Dr. Paul H. Bell, Lederle Laboratories, Pearl River, New York, U.S.A.) was also determined. The method employed was the same as for testicular hyaluronidase except that the digestion was carried out in 0·1M sodium acetate-acetic acid buffer, pH 5·5 at 25°C.

*Electrophoresis of acid mucopolysaccharides*

Before electrophoresis the acid mucopolysaccharides were prepared in the unbound form. The complexes obtained from the columns were dissolved in 60 per cent n-propanol. The polysaccharides were then precipitated free of the cetylpyridinium ion by the addition of 3 vol. of ethanol saturated with sodium acetate and dissolved in distilled water. Samples (2–5 μl.), containing approximately 2–5 μg. of acid mucopolysaccharide were subjected to electrophoresis on cellulose acetate in 0·1M phosphate buffer, pH 7·2 for 3 hours at 1mA/cm. The compounds were stained by immersing the strips in 0·04 per cent toluidine blue in 3 per cent acetic acid for 2 minutes followed by a wash in distilled water acidified with a few drops of concentrated acetic acid. The mobilities are given relative to heparin (BDH).

*Determination of hydroxyproline*

In those cases where sufficient tissue was available approximately 2 mg. of dried tumour were hydrolysed in 2 ml. of 6N HCl at 105°C. for 18 hours in sealed tubes as described by Bergman and Loxley (as yet unpublished). The hydroxyproline was estimated by the method of Neumann and Logan (1950) on 0·5 ml. aliquots of the hydrolysate, dried under vacuum and dissolved in 0.5 ml. of distilled water.

*Determination of deoxyribonucleic acid*

Samples of dried tumour (approximately 5 mg.) were extracted with 3 ml. of 5 per cent (w/v) trichloroacetic acid for 15 minutes. After centrifugation, the precipitate was re-extracted with a further 2 ml. of trichloroacetic acid under the same conditions. Following centrifugation the supernatants were combined and 2 ml. aliquots taken for the estimations. These were carried out using the diphenylamine colour reaction as recommended by Croft and Lubran (1965) for gastric wash-out specimens. Both procedures, involving the development of the colour at 30°C. and 6–13°C. for 24 and 48 hours respectively, were employed.

In practice the latter procedure was altered slightly in that the temperature of the cooler reaction was reduced to 4°C. and the development time prolonged to 96 hours. Standard DNA solution was prepared from highly polymerised calf-thymus DNA (Sigma Chemical Co. Ltd.).

### RESULTS

The acid mucopolysaccharides which were obtained from the mixed salivary tumours are shown in Table I together with their chemical characteristics. Chondroitin sulphate C was present in two fractions. The 1·5N $MgCl_2$ in 0·1M acetic acid fraction contained chondroitin sulphate C similar to that obtained from other sources, such as aorta (Antonopoulos *et al.*, 1965) and experimental granulomas (Lovell *et al.*, 1966) using the methods employed here. The compound eluted by 0·6N $MgCl_2$ was also identified as chondroitin sulphate C because it contained galactosamine and was degraded by testicular but not streptococcal hyaluronidase. Moreover, after treatment with testicular hyaluronidase the products gave the Morgan-Elson reaction (as modified by Reissig, Strominger and Leloir, 1955) for N-acetylhexosamine. According to Mathews and Inouye, (1961) this distinguishes chondroitin sulphate C from chondroitin sulphate A the breakdown products of which do not give the Morgan-Elson reaction. This chondroitin sulphate C, which differed from the other more usual form in having a reduced sulphate content and electrophoretic mobility, comprised approximately 25 per cent of the total chondroitin sulphate C in all the tumours studied regardless of the histological pattern.

The hyaluronic acid and chondroitin sulphate B, eluted by 0·3N NaCl and 1·5N $MgCl_2$ respectively, exhibited properties characteristic of these compounds. No chondroitin sulphate A, keratosulphate or heparin was found in any of the tumours.

The amounts of the individual and total mucopolysaccharides in the mixed salivary tumours are presented in Table II. The figures for chondroitin sulphate C include both fractions combined. The DNA contents, also shown in Table II, are given here as an estimate of the cellularity of the tumours. This will be equivalent to the extent of the epithelial elements as the myxomatous tissue contains relatively few cells. On a histological basis the tumours can be divided into three groups. The first, which includes cases 1–5, were predominantly myxomatous and were low in DNA. In the second group (cases 6–8) the amounts of myxomatous and epithelial tissue were approximately the same. In cases 7 and 8 the DNA levels were above those of the first group and in case 6 towards the upper limit. The third group, cases 9–13, comprised those tumours which were composed almost entirely of epithelium with only occasional small areas of myxomatous tissue. In this group the DNA levels were uniformly high.

With the exception of chondroitin sulphate B, the acid mucopolysaccharides are present in proportion to the extent of the myxomatous tissue. In all instances chondroitin sulphate C was the most abundant polysaccharide. As the tumours become more cellular the levels of chondroitin sulphate C and hyaluronic acid fall.

The level of chondroitin sulphate B was not related to those of the other mucopolysaccharides but appeared to be linked to the hydroxyproline content of the tumours (Table III). From the histology it was apparent that the levels of hydroxyproline could be related to the collagen in the capsule and septa which extended into the tumour. The presence of chondromatous tissue in the tumours, as in cases 2, 3, 5 and 7, was not associated with high levels of hydroxyproline.

There were no differences in the characteristics of the acid mucopolysaccharides obtained from tumours which arose in the submandibular region compared with those which were parotid in origin, nor in the relative proportions of the mucopolysaccharides other than in relation to their myxomatous and fibrous tissue contents.

### DISCUSSION

Three acid mucopolysaccharides, hyaluronic acid, chondroitin sulphate B and chondroitin sulphate C have been isolated from mixed salivary tumours. Chondroitin sulphate C was eluted in two fractions. This may be a somewhat

TABLE I.—*Chemical Characteristics of Acid Mucopolysaccharides Isolated from Mixed Salivary Tumours*

| Acid mucopolysaccharide | Eluant * | Hexosamine | Sulphate groups per disaccharide unit | Susceptibility to testicular hyaluronidase digestion | Susceptibility to streptococcal hyaluronidase digestion | Electrophoretic mobility (Heparin = 100) |
|---|---|---|---|---|---|---|
| Hyaluronic acid | 0.3N NaCl | Glucosamine | 0 | + | + | 50 |
| Chondroitin sulphate C | 0·6N $MgCl_2$ | Galactosamine | 0·85 | + | − | 80 |
| Chondroitin sulphate C | 1·5N $MgCl_2$ in 0·1M acetic acid | Galactosamine | 1 | + | − | 85 |
| Chondroitin sulphate B | 1·5N $MgCl_2$ | Galactosamine | 1 | − | − | 85 |

* Each eluant contained 0·05 per cent cetylpyridinium chloride.

TABLE II.—*Individual and Total Acid Mucopolysaccharide and DNA Content of Mixed Salivary Tumours*

| Case | Age (years) | Sex | Site of tumour | Acid mucopolysaccharides (μg. uronic acid/mg. dry wt.) Hyaluronic acid | Chondroitin sulphate B | Chondroitin sulphate C | Total | DNA (μg. mg. dry wt.) | Remarks |
|---|---|---|---|---|---|---|---|---|---|
| 1 | 53 | F | Parotid | 4·27 | 0·67 | 24·49 | 29·43 | 9·0 | Predominantly myxomatous |
| 2 | 37 | F | Parotid | 5·60 | 0·19 | 21·71 | 27·50 | 7·3 | ,, ,, |
| 3 | 42 | F | Submandibular | 5.76 | 0·93 | 18·64 | 25·33 | 10·4 | ,, ,, |
| 4 | 14 | M | Parotid | 2·49 | 0·40 | 23·05 | 25·94 | 14·8 | ,, ,, |
| 5 | 32 | F | Parotid | 3·93 | 0·32 | 17·82 | 22·07 | 7·6 | ,, ,, |
| 6 | 14 | M | Submandibular | 2·03 | 0·70 | 8·29 | 11·02 | 12·3 | Cellular and myxomatous |
| 7 | 28 | M | Submandibular | 3·13 | 0·71 | 12·34 | 16·18 | 17·2 | ,, ,, |
| 8 | 48 | M | Parotid | 1·34 | 0·85 | 8·81 | 11·00 | 17·5 | ,, ,, |
| 9 | 52 | F | Parotid | 0·22 | 0·92 | 1·78 | 2·92 | 24·1 | Predominantly cellular |
| 10 | 51 | F | Parotid | 0·41 | 0·89 | 5·72 | 7·02 | 22·8 | ,, ,, |
| 11 | 61 | M | Submandibular | 0·20 | 0·64 | 1·74 | 2·58 | 23·2 | ,, ,, |
| 12 | 75 | M | Submandibular | 0·58 | 0·35 | 3·27 | 4·20 | 27·0 | ,, ,, |
| 13 | 26 | F | Submandibular | 0·94 | 1·02 | 3·11 | 5·07 | 22·0 | ,, ,, |

TABLE III.—*Hydroxyproline and Chondroitin Sulphate B Content of Mixed Salivary Tumours*

| Case | Hydroxyproline (μg./mg. dry wt.) | Chondroitin Sulphate B (μg. uronic acid/mg. dry wt.) | Remarks |
|---|---|---|---|
| 9 | 18·14 | 0·92 | |
| 10 | 13·21 | 0·89 | |
| 3 | 12·06 | 0·93 | " Chondromatous " areas present |
| 7 | 11·58 | 0·71 | " Chondromatous " areas present |
| 1 | 9·25 | 0·67 | |
| 4 | 8·37 | 0·40 | |
| 2 | 5·26 | 0·19 | " Chondromatous " areas present |
| 5 | 4·67 | 0·32 | " Chondromatous " areas present |

artificial division in that the chondroitin sulphate C molecules are probably sulphated to varying extents ranging from approximately 0·8 to 1 group per disaccharide unit. This polysaccharide has thus been separated into a high and low sulphate fraction. It should be noted that the eluant for the latter, 0·6N $MgCl_2$ in 0·05 per cent cetylpyridinium chloride, will also solubilise the cetylpyridinium complex of heparin monosulphate. The two compounds can be distinguished clearly by the type of constituent hexosamine, the effect of testicular hyaluronidase and the electrophoretic mobility. The absence of any glucosamine in this fraction demonstrates conclusively that there is no heparin monosulphate present. The reduced electrophoretic mobility of this chondroitin sulphate C is probably due to its slightly lower sulphate content. This could result from the partial desulphation of normally formed chondroitin sulphate C or its incomplete synthesis.

Chondroitin sulphate B was present in greater concentration in those tumours which had well formed fibrous tissue capsules and septa. The hydroxyproline levels were given as the chemical expression of the amount of collagen present. There was good agreement between the observed amount of fibrous tissue, hydroxyproline and chondroitin sulphate B. This association between chondroitin sulphate B and mature collagen has been demonstrated previously ; *e.g.* in skin (Loewi and Meyer 1958) and experimental granulomas (Lovell *et al.* 1966). The foregoing does not exclude the possibility that the myxomatous areas contain small amounts of chondroitin sulphate B. However, the histochemical study by Azzopardi and Smith (1959), which showed the complete abolition of acid mucopolysaccharide staining after treatment with testicular hyaluronidase renders this unlikely. These workers believed however that the myxochondroid areas probably contain chondroitin sulphate A or B. The present investigation thus confirms their observations but not their suggestion as to the types of mucopolysaccharide present.

The site of origin of the tumours, whether parotid or submandibular was not associated with any particular change in the characteristics of the acid mucopolysaccharides or their relative proportions.

Although not specifically determined there were small amounts of sialic acid present in all the tumours. This conclusion was reached by the calculation of the ratios of the optical densities measured at 550 and 600 m$\mu$ in the 30° C. DNA reaction. The ratios were slightly below those of pure DNA, indicating the presence of sialic acid (Croft and Lubran 1965). The DNA estimations were made quantitative by carrying out the reaction at 4° C., at which temperature there is no interference from sialic acid.

### SUMMARY

Mixed salivary tumours have been found to contain hyaluronic acid, chondroitin sulphate B and chondroitin sulphate C. The myxomatous areas are believed to comprise predominantly chondroitin sulphate C, which may be divided into two fractions dependent on the degree of sulphation, and hyaluronic acid. The chondroitin sulphate B is probably associated with the mature collagen of the capsule and septa.

We are grateful to Professor R. C. Curran for his advice and encouragement and to Miss V. Hark for her technical assistance. The work was supported by the University of London Central Research Fund and the Medical Research Council.

### REFERENCES

Antonopoulos, C. A. and Gardell, S.—(1963) *Acta chem. scand.*, **16**, 1521.
Antonopoulos, C. A. Gardell, S. and Hamnstrom, B.—(1965) *J. Atheroscler. Res.*, **5**, 9.
Antonopoulos, C. A., Gardell, S., Szirmai, J. A. and Tyssonsk, E. R. D.—(1964) *Biochim. biophys. Acta*, **83**, 1.
Azzopardi, J. G. and Smith, O. D.—(1959) *J. Path. Bact.* **77**, 131.
Croft, D. N. and Lubran, M.—(1965) *Biochem. J.*, **95**, 612.
Grishman, E.—(1952) *Cancer, N.Y.*, **5**, 700.
Hemplemann, L. H. and Womack, N. A.—(1942) *Ann. Surg.*, **116**, 34.
Loewi, G. and Meyer, K.—(1958) *Biochim. biophys. Acta*, **27**, 453.
Lovell, D., Schorah, C. J. and Curran, R. C.—(1966) *Br. J. exp. Path.*, **47**, 228.
Mathews, M. B. and Inouye, M.—(1961) *Biochim. biophys. Acta*, **53**, 509.
Neumann, R. E. and Logan, M. A.—(1950) *J. biol. Chem.*, **184**, 299.
Reissig, J. L., Strominger, J. L. and Leloir, L. F.—(1955) *J. biol Chem.*, **217**, 959.
Yates, P. O. and Paget, G. E.—(1952) *J. Path. Bact.*, **64**, 881.